# Prevalence of human pathogenic *Yersinia enterocolitica* in Swedish pig farms

**DOI:** 10.1186/s13028-018-0393-5

**Published:** 2018-06-25

**Authors:** Therese Råsbäck, Thomas Rosendal, Michael Stampe, Axel Sannö, Anna Aspán, Katarina Järnevi, Elina Tast Lahti

**Affiliations:** 1 Department of Microbiology, National Veterinary Institute (SVA), 751 89 Uppsala, Sweden; 2 Department of Disease Control and Epidemiology, National Veterinary Institute (SVA), 751 89 Uppsala, Sweden; 3 Farm and Animal Health, Kungsängens Gård 6B, 753 23 Uppsala, Sweden; 4 Department of Clinical Sciences, Swedish University of Agricultural Sciences (SLU), Box 7070, 750 07 Uppsala, Sweden

**Keywords:** Biotyping, Farm, Mass spectrometry, Pig, Prevalence, Risk factors, Serotyping, *Yersinia enterocolitica*, Zoonosis

## Abstract

**Background:**

Pigs are the most important reservoir for human pathogenic *Yersinia enterocolitica*. We investigated the herd prevalence of human pathogenic *Y. enterocolitica* in Swedish pig farms by analysing pen faecal samples using a cold enrichment of 1 week and thereafter subsequent plating onto chromogenic selective media (CAY agar).

**Results:**

Pathogenic *Y. enterocolitica* was found in 32 (30.5%) of the 105 sampled farms with finisher pigs. Bioserotype 4/O:3 was identified at all but one farm, where 2/O:9 was identified. Pen-prevalence within the positive herds varied from 1/4 to 4/4 pens. The calculated intra-class correlation coefficient ICC (0.89) from a model with a random effect for grouping within herd indicated a very high degree of clustering by herd. None of the explored risk factors, including herd size, herd type, pig flow, feed type, access to outdoors, evidence of birds and rodents in the herd, usage of straw, number of pigs in sampled pen and age of pigs in pen were significantly associated with *Y. enterocolitica* status of the pen. The use of high pressure washing with cold water was significantly associated with *Y. enterocolitica* in the pen (OR = 84.77, 4.05–1772). Two culture methods were assessed for detection of *Y. enterocolitica*, one of which included the use of a chromogenic agar (CAY agar) intended for detection of human pathogenic *Y. enterocolitica*. The chromogenic media was found equal or superior to traditional methods and was used in this study. The isolates obtained were characterised by biotyping, serotyping, mass spectrometry (MALDI-TOF) and PCR. Characterisation by MALDI-TOF gave identical results to that of conventional bioserotyping. All porcine isolates were positive for the *ail* and *inv* genes by PCR, indicating that the isolates were most likely pathogenic to humans.

**Conclusions:**

Human pathogenic *Y. enterocolitica* was found in nearly one-third of the Swedish pig farms with finisher pigs. The use of high pressure washing with cold water was associated with the presence of *Y. enterocolitica* in the pen. A modified culturing method using a chromogenic agar was efficient for detection of pathogenic *Y. enterocolitica* in pig faeces. The use of masspectrometry for identification and subtyping was in agreement with conventional biotyping and serotyping methods.

**Electronic supplementary material:**

The online version of this article (10.1186/s13028-018-0393-5) contains supplementary material, which is available to authorized users.

## Background

Yersiniosis caused by *Yersinia enterocolitica* is one of the most reported zoonoses in the EU [1] with pigs being the most important reservoir [2–4]. In humans, *Y. enterocolitica* causes gastroenteritis whereas the infection in pigs is asymptomatic [3, 5, 6]. Most patients recover fully but the infection can lead to complications, such as septicaemia, reactive arthritis or erythema nodosum [5–8].

The incidence of yersiniosis is higher in the north-eastern Europe [1] compared to the rest of the European continent. In Sweden, the incidence rate is 2.27–6.18 cases per 100,000 inhabitants [9]. However, the true incidence in Sweden is estimated to be 7.7 times higher [10]. In Sweden, pathogenic *Y. enterocolitica* has previously been detected in fattening pigs [11], in pigs at slaughter [12], and in wild boars [13], but the prevalence in pig farms has not been investigated.

*Yersinia enterocolitica* is a heterogeneous species divided into six biotypes and several serotypes [14]. Bioserotype 4/O:3 is the most frequent bioserotype in pigs and humans [3, 4], whereas biotype 1A is considered non-pathogenic [15]. Detection of pathogenic *Y. enterocolitica* in non-human samples is time consuming and laborious; a cold enrichment step is needed [16]. A chromogenic culture media, CHROMagar™ *Y. enterocolitica,* has been suggested to reduce workload and cost in detection [17–19].

Pigs acquire the infection at farms either from the environment or via the sows and start shedding the organism in faeces from the age of 14 weeks [20–22]. At slaughter age, however, most pigs no longer shed the bacterium in faeces but *Y. enterocolitica* can be frequently isolated in the tonsils [23, 24]. Pig carcasses and thus pork meat become contaminated at abattoirs by contamination from the oral cavity and intestinal contents [5, 20].

The aim of this study was to estimate the prevalence of pathogenic *Y. enterocolitica* in Swedish pig farms. We further assessed a detection method and compared characterisation by MALDI-TOF to conventional biotyping and serotyping.

## Methods

### Assessment of culture method for detection of *Yersinia enterocolitica* in pig faeces

For enumeration, two broths prepared in-house were used: phosphate-buffered saline containing 2% sorbitol and 0.15% bile salts (PSB) and phosphate-buffered saline with 0.5% peptone, 1% mannitol and 0.15% bile salts (PMB). Five microliter of colony material were picked from a *Y. enterocolitica* of bioserotype 2/O:9 (Culture Collection University of Göteborg, Sweden, CCUG 8239) and a *Y. enterocolitica* of bioserotype 4/O:3 (National Food Agency, Uppsala, Sweden, SLV412) culture grown overnight on blood agar plate (Blood agar Base no2, Oxoid) with 5% horse blood, inoculated into 5 mL of broth, PSB and PMB, respectively, and carefully mixed. A serial dilution was performed by transferring 0.5 mL of inoculated broth of PSB and PMB, respectively, into 4.5 mL non-inoculated broth to a final dilution of 10^−9^. Colony count was assessed on by plating 100 µL broth from all dilutions using Cefsulodin Irgasan Novobiocin (CIN) (Oxoid, Basingstoke, UK) and CAY plates (CHROMagar™ *Y. enterocolitica*, CHROMagar, Paris) in parallel; plates were incubated at 37 °C for 24–48 h.

Faecal material from late stage finisher pigs negatively tested for *Y. enterocolitica* by culture was spiked with *Y. enterocolitica* CCUG 8239 and SLV412. This experiment was completed by, in parallel, mixing 3 g of faeces with 3 mL inoculated PSB and PMB broth from a serially inoculated broth dilution, and further diluting the mixtures 1:10 in PSB and PMB respectively. The inoculated broth cultures and faeces mixtures were then incubated at 4 °C for 7–8 days. The PSB mixtures were cultured according to a modified Nordic Committee on Food Analysis (NMKL) method no. 117 [25] and the PMB mixtures using a comparative method [21].

In the modified NMKL method, after cold enrichment, 0.1 mL of the PSB mixture was transferred to 10 mL of in-house prepared Modified Rappaport broth (MRB) and incubated at 22 °C for 4 days. A loopful (10 µL) of the enriched broth was streaked onto CIN and CAY agar plates, in parallel. The plates were further incubated at 30 °C for 24–48 h.

In the comparative method, 10 µL of the PMB mixture was streaked onto CIN and CAY agar plates. The plates were incubated at 25 °C for 24–48 h [17, 21].

Suspected *Y. enterocolitica* colonies were streaked onto a blood agar plate, incubated at 25 °C for 24 h and confirmed as *Y. enterocolitica* using Matrix-Assisted Laser Desorption Ionization Time-of-Flight Mass Spectrometry, MALDI-TOF (Bruker Daltonics, Bremen, Germany) according to the manufacturer’s instructions.

A total of 82 strains of *Y. enterocolitica* were streaked onto CAY agar plates and incubated at 25 °C for 24–48 h (Table 1). Fifty of these strains were kindly provided by the Public Health Agency of Sweden, originally isolated on CIN agar plates and previously described [26], two were of bioserotype 4/O:3 (SLV412 and SLV413) and obtained from the National Food Agency, Sweden and one was a reference strain (CCUG 8239). Plates with uncharacteristic colony morphology were further sub-cultured at 28 and 30 °C, respectively, for 24–48 h. All strains, except for those of biotype 1A, were further cultured on tryptone soya agar (TSA, Oxoid) and incubated at 22 °C for 48 h before bioserotyping by MALDI-TOF [26].

**Table 1:** Phenotypic characterisation of *Yersinia enterocolitica* isolates obtained from diarrhoeic patients (n = 50) and finisher-pig farms (n = 32)

Origin	Farms (no.)/patients (no.)	Bioserotype	Colony colour on CAY		Bioserotype as determined by MALDI-TOF	Aesculin test
			After 24 h	After 48 h		
Porcine	31	4/O:3	White/mauve	Mauve	4/O:3	N/A
Porcine	1	2/O:9	White/mauve	Mauve	2/O:9	N/A
Human	12	O:3	White/mauve	Mauve	4/O:3	N/A
Human	9	1A	Blue	Blue opaque	N/A	N/A
Human	7	O:3	White	Mauve	4/O:3	N/A
Human	7	O:9	White/mauve	Mauve	2/O:9	N/A
Human	4	O:8	Blue	Blue opaque	Biotype 1A*	+
Human	4	O:21	Blue	Blue opaque	1A/O:21	N/A
Human	3	O:3	White	White	4/O:3	−
Human	2	O:8	White/poor growth	Mauve	Biotype 1B*	−
Human	2	O:5/27	White/mauve	Mauve	2/O:27	N/A

Bioserotypes for human strains were provided by the National Food Agency in Sweden

N/A, not available; +, positive; −, negative

* Aesculin test was used to differentiate between biotype 1A and 1B, if subtyping could not be obtained by MALDI-TOF alone

### Selection of herds

In 2014–2015, the total number of pig herds in Sweden was 1228 [27]. The veterinary service company, Swedish Farm and Animal Health, supplies animal health service to a majority of these herds. Pig herds slaughtering more than 1300 pigs annually and served by Farm and Animal Health were eligible for inclusion in the study. A total of 105 pig herds were selected using simple random sampling. The sample size was calculated based on estimates of the expected herd-prevalence of 40% (CI 95%), within-herd prevalence of 50% (CI 95%) as well as an expected test sensitivity of 80% (CI 95%). The sampling aimed to estimate the prevalence at the herd level to within 10% of the true prevalence. Based on these assumptions, and adjusting for a finite population of 1000 herds, the target sample size was 103 herds using methods previously presented [28].

### Sampling of pig herds

A total of 105 farms with finisher pigs were sampled between September 2014 and January 2015. Sampling was completed Mondays through Thursdays. The farms were visited by a pig veterinarian from Farm and Animal Health. On each farm, one unit with finisher pigs was selected for sampling, preferably with pigs of 16–24 weeks of age. A unit was defined as a group of pigs kept within one building and intended to be slaughtered at same time. Within one unit, pigs were kept in pens with 8–12 pigs per pen. However, on farms where pigs had outdoor access, the group size ranged from 10 to 30 pigs. On farms with several units of pigs within this age range, a unit with pigs closest to the age of 24 weeks was chosen. A pooled faecal sample was collected from the floor of each of four pens per farm. During the visit, the veterinarian filled in a questionnaire about the farm production system. The questionnaire was administered using the *sdaps* framework (http://sdaps.org/) and consisted of questions about animal flow, cleaning, feeding, rodent and bird control, flooring types and use of straw. The questionnaires are attached as Additional files 1 and 2. For each of the four sampled pens, the number of pigs, the age in weeks and an indication of the amount of straw used in the pen was also recorded. The samples, along with the questionnaire, were delivered by mail and within 3 days to the National Veterinary Institute (SVA), Uppsala, Sweden.

### Detection of *Y. enterocolitica* in samples collected from pig farms

The analysis of the samples was started within 8 h of arrival to the laboratory. A homogenised faecal sample (5 g) was mixed with 50 mL PMB broth and incubated at 4 °C for 7–8 days. A loopful (10 µL) from the top layer of the homogenate was streaked onto a CAY plate and incubated at 25 °C for 24–48 h. Typical colonies (white, moist, smooth, round colonies of 1–2 mm with or with a tendency of “bull’s eye” surrounded by transparent area) were picked after 1 day of incubation and subcultured onto blood agar for 24 h at 30 °C. Colonies white on CAY after 48 h and positively tested with oxidase (Bactidrop™, Oxoid) were discarded. Colonies changing colour from white to mauve (pink/lilac) on CAY plates after an incubation of 48 h and non-haemolytic on horse blood agar were analysed to species level by MALDI-TOF. Isolates confirmed as *Y. enterocolitica* were stored at − 70 °C.

### Characterisation by MALDI-TOF subtyping, and conventional biotyping and serotyping

For subtyping with MALDI-TOF, isolates of *Y. enterocolitica* were streaked onto tryptone-soya-agar (TSA) plates without blood and incubated at 22 °C for 24 h. Thereafter, a protocol for MALDI-TOF subtyping was followed [26]. For conventional biotyping and serotyping, these plates were incubated for 48 h. Biotyping was performed according to ISO/TC 34/SC 9 N where hydrolysation of aesculin as well as production of xylose, pyrazinamidase, lipase, trehalose, and indole were tested. Serotyping was performed and according to the manufacturer’s instructions as slide agglutination using diluted *Y. enterocolitica* antisera O3 and O9 (Reagensia AB, Solna, Sweden). Sodium chloride solution was used as a negative control.

### PCR analysis

A real-time PCR targeting for the chromosomally encoded attachment and invasion (*ail*) gene was used [29]. Primers and a TaqMan MGB probe were purchased from Eurofins MWG Operon, Germany (Table 2). The protocol [29] was used with following modifications: PCR was performed in 15 µL reaction volumes containing 2× PerfeCTa qPCR Toughmix with Low ROX (Quanta Biosciences, Gaithersburg, MD), 500 nM of each primer, 100 nM of probe and 2 µL template. The PCR was performed in an ABI 7500 Fast thermal cycler (Applied Biosystems, Foster City, CA) with the following temperature profile: 95 °C for 3 min followed by 40 cycles of 95 °C for 3 s and 60 °C for 30 s at which fluorescence was measured. An internal positive control (IPC) was added to every PCR using a commercially available TaqMan exogenous IPC kit (Life Technologies, USA). The reagent kit included primers, a VIC probe, IPC target DNA and blocking solution. The IPC target DNA was diluted × 50 to enable an expected Cycle threshold (Ct) for the IPC within the range 35–38. To the diluted kit reagent, 6 μL ddH_2_O and 5 μL template was added. If the IPC Ct was > 38 and no Ct was detected for the bacterial target gene, the template was diluted 1:10 with ddH_2_O and subjected to a second PCR. A Ct ≤ 40 was considered a positive result [13].

**Table 2:** Primer sequences

qPCR	Primer name	Primer sequence	Amplicon (bp)
*Y. enterocolitica*, *ail*-gene [29]	Forward primer:	CCCAGTAATCCATAAAGGCTAACATAT	163
	Reverse primer:	ATGATAACTGGGGAGTAATAGGTTC	
	Probe: (FAM-MGB prob)	TGACCAAACTTATTACTGCCATA	
Genus *Yersinia*, *inv*-gene [30]	Forward primer:	TTGACACAACCTTAGGCAATATGG	73
	Reverse primer:	ACTGGTCAATGGTGCGCTATAA	
	Probe: (FAM-MGB prob)	CGTTATCACGGATCACAATGACGGCA	

Also, a *Yersinia* genus specific PCR targeting the *inv* gene was used for screening the isolates [30].

### Risk factor analysis

The correlation between production practices recorded on the survey at the pen and herd levels and the odds of detection of *Y. enterocolitica* in the collected faecal samples was tested by mixed variable logistic regression in the *lme4* package (version 1.1–12) for *R* (version 3.2.2). All interpreted models included a random intercept term (u_herd_) to account for the repeated pen measurements within each herd. The intra-class correlation coefficient (ICC) was also calculated $\left( {ICC = \frac{{\sigma_{herd}^{2} }}{{\left( {\sigma_{herd}^{2} + \pi^{2} /3} \right)}}} \right)$ from an empty model: $logit\left( {p_{i} } \right) \sim \beta_{0} + u_{herd\left( i \right)}$.

## Results

### Assessment of culture methods

The broth dilutions yielded detectable growth up to 10^−8^ (4–7 colonies) on CAY plates. On CIN plates, *Y. enterocolitica* was detected up to 10^−8^ using PMB broth (4 colonies) and up to 10^−7^ using PSB broth (15 colonies). In spiked faecal samples, *Y. enterocolitica* was detected up to a dilution of 10^−9^ when using PMB and up to 10^−8^ when using PSB.

**Fig. 1:**
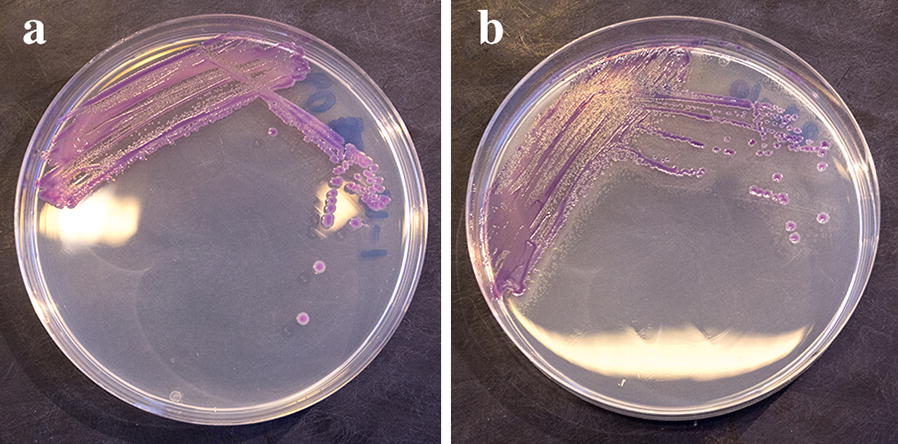
**a** *Yersinia enterocolitica* bioserotype 2/O:9 isolate, displaying pink colour with a mauve coloured “bull´s eye”, on CHROMagar™ *Y. enterocolitica* (CAY) plate incubated at 25 °C for 24–48 h. **b** Swarming colonies of a *Yersinia enterocolitica* bioserotype 1B/O:8 isolate on CHROMagar™ *Y. enterocolitica* (CAY) plate incubated at 25 °C for 24–48 h

**Fig. 2:**
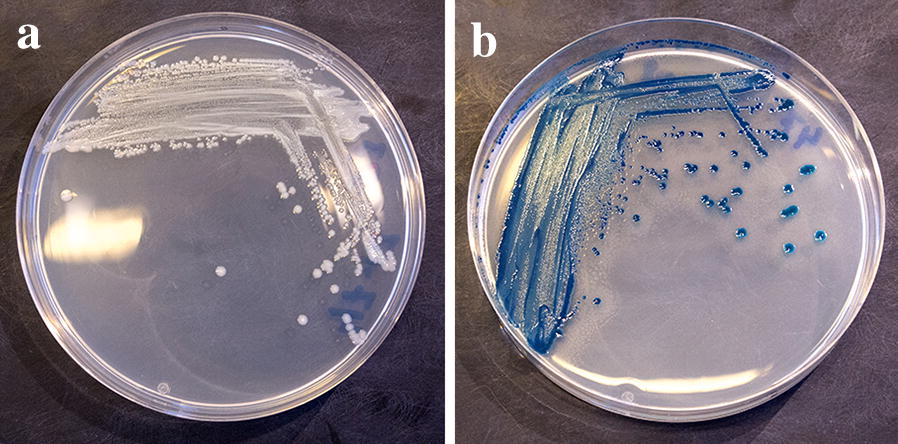
**a** *Yersinia enterocolitica* bioserotype 4/O:3 isolate on CHROMagar™ *Y. enterocolitica* (CAY) plate incubated at 25 °C for 24–48 h. White colony formations with a transparent “bull’s eye”. **b** *Yersinia enterocolitica* bioserotype 1A/O:8 isolate on CHROMagar™ *Y. enterocolitica* (CAY) plate incubated at 25 °C for 24–48 h. Blue swarming colonies with dark metallic blue “bull’s eye”

On CAY agar, colonies of the clinical *Y. enterocolitica* isolates were either smooth or with a tendency of swarming seen as a thin haze (Fig. 1) except for one strain poorly growing on CAY agar (Table 1). Colonies of this and another clinical isolate had only a tendency of pink/mauve colour after 48 h incubation at 25 °C (Table 1). However, colonies of three other strains of bioserotype 4/O:3 were white with a transparent “bull’s eye” at all incubation temperatures tested (Fig. 2). Colonies of 17 clinical strains were swarming and dark metallic blue in colour (Fig. 2).

### Pig farms

**Fig. 3:**
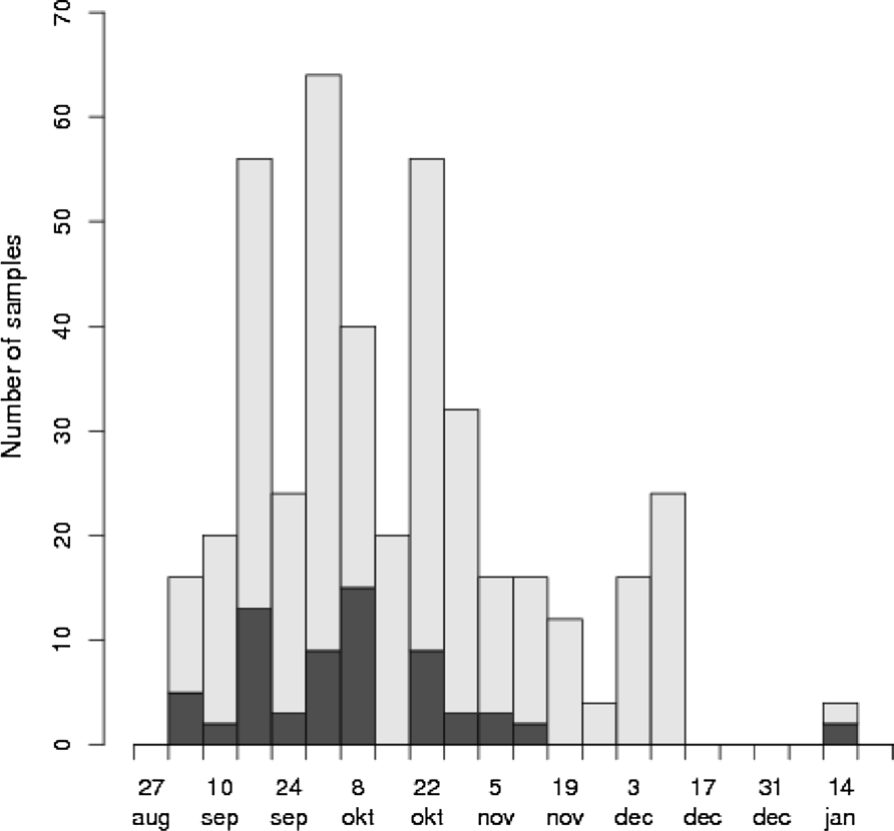
Number of pig herds sampled per month. Black cells in the diagram represent pen samples where *Y. enterocolitica* was isolated; grey cells represent negative samples

Sampling from 105 pig farms with finisher pigs was performed between September 2014 and January 2015 (Fig. 3). The farms sampled, were from 14 Swedish counties, and the number per county was calculated to represent the non-uniform distribution of Swedish pig farms. An additional map file shows the geographical distribution (Additional file 3). Fifty-two (49.5%) of the farms were specialised finisher herds, 46 (43.8%) were farrow to finish production units of which 19 (18.1%) were satellites of sow pools, six (5.7%) were breeding stock suppliers and one (0.95%) was a centre of a sow pool. Sow pool production constitutes a central herd that services and leases out pregnant sows for farrowing to satellite herds. Piglets are kept and raised at the satellite herds while the sows return to the central unit after weaning. Among the farms sampled, the average number of finisher pigs produced per year was 5649 (range 1346–39,940). Farms specialised in finisher production had an average of 6353 pigs slaughtered per year, which is slightly larger than the Swedish national average. Of the integrated farms, the average number of sows was 293 (range 45–1100).

The management of the sampled farms varied. Of the 105 farms, two farms kept finisher pigs with access to outdoors. Only five farms reported feeding finisher pigs a dry ration, the remaining used a liquid feeding system. Eighteen of the farms reported using a complete feed ration for finisher pigs and the remaining farms used their own produced or purchased grain with a broad range of other feed ingredients including: distiller’s grains, whey, soy beans, potatoes, milk, by-products of pasta production and bread.

The presence of straw assessed ‘at least visible’ in the finisher pens was reported for all but four farms and most of the pens were reported to have ‘plenty’ of straw. Partially slatted concrete flooring was used in all but the two farms with outdoor access in which deep straw bedding was used. Ninety of the farms reported emptying the barn between all batches of pigs and 12 reported ‘almost always’ emptying the barn between batches. Thirty-seven farms reported using mechanical scraping between batches of pigs, 96 using high pressure cleaning with hot or cold water and detergent, 43 farms used a disinfectant after cleaning and 35 farms reported including a drying period after cleaning and before adding new pigs to the barn.

### *Yersinia enterocolitica* in pig farms

*Yersinia enterocolitica* was detected from 32 (30.5%) of the 105 sampled farms, and a total of 92 porcine isolates were obtained. All four pen samples were positive at 14 of the 32 farms, at four farms three pen samples were positive, at nine farms two and at five farms only one pen sample was positive. *Y. enterocolitica* was detected throughout the sampling period. Bioserotype 4/O:3 was identified at all but one farm, where 2/O:9 was identified. Only one bioserotype was identified per farm.

All porcine isolates were characterised with conventional biotyping and serotyping methods and subtyped by MALDI-TOF, whereas all human strains not already determined to be biotype 1A were subtyped by MALDI-TOF only. Bioserotyping with conventional methods and subtyping by MALDI-TOF gave identical results (Table 1). The MALDI-TOF method could subtype all porcine isolates and all but six of the human strains; these had to be further assessed for hydrolysis of aesculin to discriminate between biotype 1A and 1B [26]. All porcine isolates gave a positive signal in the PCR for the *Yersinia* genus specific assay as well as in the PCR for the virulence gene (*ail*) assay; the control strain of *Y. pestis* was positive only in the *Yersinia* genus PCR.

### Risk factor analysis

None of the explored risk factors, including herd size, herd type, pig flow, feed type, access to outdoors, evidence of birds and rodents in the herd, usage of straw, number of pigs in sampled pen and age of pigs in pen were significantly associated with *Y. enterocolitica* status of the pen. The use of high pressure washing with cold water was significantly associated with *Y. enterocolitica* in the pen [OR = 84.77, (4.05–1772)] after adjusting for other washing methods used in the herd. The largest proportion of the variance of pen level *Y. enterocolitica* status was at the herd level (ICC = 0.89) indicating that *Y. enterocolitica* is clustered in herds. Detailed summary of non-significant findings and tabulation of the *Y. enterocolitica* status of herds by the measured variables are included in Additional files 4 and 5).

## Discussion

To identify cost effective measures to reduce the incidence of human yersiniosis, the prevalence and epidemiology within the pig farms needs to be understood. Current knowledge is insufficient regarding the incidence, possible geographical differences and other differences in herd-prevalence or risk factors.

The pig herds included in this study represented the general geographical distribution of pig herds in Sweden, with the majority of pig herds in the southern part of the country. Overall, pathogenic *Y. enterocolitica* was detected in 30.5% of the pig herds. This is less than in many previous studies in other EU countries where herd-prevalences of 69–100% have been described when faecal samples have been tested [14]. However, the peak in faecal excretion is at 2–5 months [22–24, 31] and pigs in our study were of the age of 4–6 months and thus the prevalence may be underestimated. Also, a combination of more than one enrichment steps might have resulted in higher prevalence [32].

The pen-prevalence within the positive herds varied from 1/4 to 4/4 pens. The calculated ICC (0.89) from the model with a random effect for grouping within herd indicated a very high degree of clustering by herd. This indicates that the factors affecting *Y. enterocolitica* status in these herds are at the herd level, not factors that vary by pen or animal within herds. The finding that the use of high pressure washing with cold water was a risk factor for *Y. enterocolitica* is perhaps counterintuitive. However, a study of cleaning practices in Ontario, Canada pig herds showed that cleaning with cold water was associated with *Salmonella* shedding [33]. It is reasonable to consider the cleaning variables together as a set of practices at the herd and not as independent management factors. This may explain the increased risk of *Yersinia* when washing with cold water as it could be simply an indication that the herd does not use another set of washing procedures that are protective but not recognisable in the current study. The fact that no other significant hygiene or other herd level risk factors were identified, despite the high within-herd clustering of the outcome may be related to the questions asked on the survey being related to the finisher phase of production only. In other studies, proper disinfection routines have been protective [34]. If finisher herds become infected from their source herds, then the important risk factors for *Y. enterocolitica* status would be found at the nursery or farrowing herds and would therefore result in the high degree of clustering of the outcome within herd (ICC = 0.89). To understand this potential spread from earlier stages of production to the finisher phase, a longitudinal investigation of *Y. enterocolitica* positive finisher herds and their source nurseries and sow herds that includes molecular typing of the bacterial isolates would be necessary. Further studies are also needed to establish within herd prevalence, as four pen samples were too few to accurately calculate this.

Cold enrichment is widely used for detection of *Y. enterocolitica* from clinical, food, and environmental samples [16]. In this study, a cold enrichment step of 1 week was applied. Attempts to shorten the culturing steps have given conflicting results. Cold enrichment of 1 week was as sensitive as longer cold enrichment steps [25]. Shorter cold enrichment steps have also been applied [35]. However, cold enrichment of 14 days was superior to 7 days in isolating pathogenic *Y. enterocolitica* [36]. Also, using a detection method with cold enrichment periods of 7 and 14 days in combination resulted in more isolations [32] applying more than one enrichment step could have increased the prevalence estimate of our study. However, further subtyping by MALDI-TOF was compared with conventional bioserotyping for a faster and less expensive characterisation of isolates. Further, subtyping by MALDI-TOF was compared with conventional bioserotyping for a faster and less expensive characterisation of isolates.

In this study, PMB seemed to be slightly more sensitive than PSB in detection of *Y. enterocolitica* in broth and spiked pig faeces. However, as less than five colonies were detected on plates of the 10^−9^ dilution the differences between the two methods were not tested with statistical methods. We further tested a chromogenic selective medium, CAY agar, to enable an easier detection of the bacterium. To the authors’ knowledge, this plate has not been tested for detection of *Y. enterocolitica* in porcine faecal samples, but promising results were obtained in a study of human stool samples [18]. Compared to the CIN medium, the use of CAY plates further improved the detection of suspected pathogenic *Y. enterocolitica* colonies from pig faeces as the *Y. enterocolitica* colonies were easier to identify on CAY agar plates most likely due to a suppression of competing microbial population. However, a comparable, high recovery rate was obtained using both methods.

Colonies of three clinical *Y. enterocolitica* strains and the frequently subcultured CCUG strain used at our laboratory did not change colour to mauve on the CAY plates but remained white even when incubated at 30 °C, which contrasts with the findings of other authors [17, 18, 37]. Frequent subculturing might have resulted in the atypical colony colour, as a less subcultured CCUG strain obtained from the frozen culture stock had mauve colonies (data not included).

Bioserotype 4/O:3 was the most common bioserotype on Swedish pig farms as it was detected from all farms except for one where bioserotype 2/O:9 was found. Bioserotype 4/O:3 is the most common bioserotype in pigs in several European countries [1, 38–40]. Only one bioserotype was detected on each farm, indicating that few bioserotypes are circulating within the Swedish pig farms. All porcine isolates had the *ail* gene for pathogenicity, confirming pathogenicity of all isolates.

## Conclusions

Human pathogenic *Y. enterocolitica* was found in nearly one-third of the Swedish pig farms with finisher pigs, which confirms the importance of pigs as a reservoir for this pathogen. In order to decrease the public health risk, cost-efficient methods to control the infection in the pig reservoir are needed.

## Additional files

**Additional file 1.** A questionnaire with questions on the farm management system and filled in by the veterinarian.

**Additional file 2.** The questionnaire translated to English.

**Additional file 3.** A map showing the pig herds sampled per county. The distribution of samples reflects the non-uniform distribution of Swedish pig farms.

**Additional file 4: Appendix Table S1.** The proportion of herds positive for *Yersinia enterocolitica* for each of the categorical variable levels recorded in the questionnaire. The *P*-values and associated odds ratios for the association of each variable with pen level *Y. enterocolitica* status, tested by logistic regression controlling for repeated pen measurements within herd by a random effect.

**Additional file 5: Appendix Table S2.** A summary of the continuous variables recorded in the questionnaire and the *P*-values and the associated odds ratios for the association of each variable with pen level *Y. enterocolitica* status, tested by logistic regression controlling for repeated pen measurements within herd by a random effect.
