# Supplementary material for: Prevalence of human pathogenic Yersinia enterocolitica in Swedish pig farms
Source: Acta Vet Scand. 2018 Jun 25;60:39. doi: 10.1186/s13028-018-0393-5 (PMC6020225; doi:10.1186/s13028-018-0393-5)
Supplement: Supplementary file 1 — Additional file 1. A questionnaire with questions on the farm management system and filled in by the veterinarian. [file 13028_2018_393_MOESM1_ESM.pdf]

Additional file 1. A questionnaire with questions on the farm management system and filled in by the veterinarian.

Statens Veterinärmedicinska Anstalt
Studie om förekomsten av Yersinia enterocolitica i grisbesättningar

This questionnaire is automatically read by a computer program. Please use a pen for filling in your answers.
Check: ☒ You can check any number of boxes in selection questions.
Uncheck to correct: ■ For questions with a range (1–5) choose the answer the mark that fits best.

Följ bifogade provtagningsinstruktion, och fyll i nedanstående frågor om besättningen.**Sida 1 och 2.**

Gårdens löpnummer:

1 Frågor om besättningen

1.1 Töms slaktgrisavdelningen mellan omgångar?

- ☐ Alltid
- ☐ Nästan alltid
- ☐ Oftast
- ☐ Aldrig/Sällan

1.2 Vilka metoder används vid rengöring av slaktgris-stallet?(Du kan välja ett eller flera alternativ)

- ☐ Mekanisk rengöring
- ☐ Rengöring med högtryck (kallt vatten)
- ☐ Rengöring med högtryck (varmt vatten)
- ☐ Rengöring med högtryck (med rengöringsmedel)
- ☐ Desinfektionmedel används
- ☐ Torrperiod efter rengöring eller desinfektion

Hur många dagar är torrperioden?

Annat:

1.3 Har slaktgrisarna tillgång till utevistelse?

- ☐ Ja
- ☐ Nej

1.4 Vilken typ av foder ges till slaktgrisarna?

- ☐ Torr foder
- ☐ Blötfoder

1.5 Vilket eller vilka val beskriver bäst fodret? (Du kan välja ett eller flera alternativ)

- ☐ Färdigfoder
- ☐ Koncentrat och Spannmål
- ☐ Vassle tillsätts
- ☐ Vatten tillsätts

Annan beskrivning:

1.6 Finns det ett gnagarkontrollprogram?

- ☐ Ja
- ☐ Nej

1.7 Ser du spillning eller märken av gnagare i stallet?

- ☐ Alltid
- ☐ En gång i veckan
- ☐ En gång i månaden
- ☐ Aldrig/Sällan

1.8 Ser du fåglar, eller spillning/märken av fåglar i stallet?

- ☐ Alltid
- ☐ En gång i veckan
- ☐ En gång i månaden
- ☐ Aldrig/Sällan

1.9 Vilken typ av golvmaterial finns i slaktgrisboxarna?

- ☐ Helgolv med skrapgång
- ☐ Helgolv med spaltgolv på samma höjd
- ☐ Helgolv med nedsänkt spaltgolv
- ☐ Helgolv med upphöjt spaltgolv
- ☐ Djupströbädd
- ☐ Utegrisar

Annat:

1.10 Vilket strömedel används?

- ☐ Halm
- ☐ Spån
- ☐ Torv
- ☐ Torvmix
- ☐ Halmspånpellets

Annat:

2 Frågor om varje provtagen box

Box 1

2.1 Syns strö i boxen?

- ☐ Rikligt
- ☐ Spår av strö
- ☐ Nästan inte alls

2.2 Hur många grisar finns i boxen?

2.3 Hur gamla (antalet veckor) är grisarna i boxen?

Box 2

2.4 Syns strö i boxen?

- ☐ Rikligt
- ☐ Spår av strö
- ☐ Nästan inte alls

2.5 Hur många grisar finns i boxen?

2.6 Hur gamla (antalet veckor) är grisarna i boxen?

Box 3

2.7 Syns strö i boxen?

- ☐ Rikligt
- ☐ Spår av strö
- ☐ Nästan inte alls

2.8 Hur många grisar finns i boxen?

2.9 Hur gamla (antalet veckor) är grisarna i boxen?

Box 4

2.10 Syns strö i boxen?

- ☐ Rikligt
- ☐ Spår av strö
- ☐ Nästan inte alls

2.11 Hur många grisar finns i boxen?

2.12 Hur gamla (antalet veckor) är grisarna i boxen?
