# Supplementary material for: Prevalence of human pathogenic Yersinia enterocolitica in Swedish pig farms
Source: Acta Vet Scand. 2018 Jun 25;60:39. doi: 10.1186/s13028-018-0393-5 (PMC6020225; doi:10.1186/s13028-018-0393-5)
Supplement: Supplementary file 2 — Additional file 2. The questionnaire translated to English. [file 13028_2018_393_MOESM2_ESM.docx]

Additional file 2. The questionnaire translated to English.

1.1 Is the finisher unit emptied of pigs completely between batches?

- Always

- Almost always

- Most often

- Never/Rarely

1.2 Which methods are used to clean the finisher unit? (You may select multiple options)

- Mechanical cleaning (scraping of floor)

- High pressure washing with cold water

- High pressure washing with hot water

- High pressure washing with an added detergent

- Disinfectant

- Drying period after cleaning or disinfection

How long in days is the drying period_______________

Other: _______________

1.3 Do the finisher pigs have access to the outdoors

- Yes

- No

1.4 What type of feed do the finisher pigs receive?

- Dry feed

- Wet feed

1.5 Which best describes the finisher ration? (You may select more than one option)

- Complete commercial feed

- grain with feed concentrate

- Added whey

- Added water

Other description: _______________________

1.6 Does the herd have a rodent control programme?

- Yes

-No

1.7 Do you see evidence of rodent droppings or marking in the barn?

- Always

- Once a week

- Once a month

- Never/Rarely

1.8 Do you see birds, bird droppings or evidence of birds in the barn?

- Always

- Once a week

- Once a month

- Never/Rarely

1.9 Which type of flooring is in the finisher pens?

- Solid flooring with a gutter

- Solid flooring with a section of slatted flooring at the same height

- Solid flooring with a sunken section of slatted flooring

- Solid flooring with a raised section of slatted flooring

- Deep litter

- Outdoor pen

Other: __________________________

1.10 Which type of recreational bedding material is used?

- Straw

- Wood shavings

- Peat

- Peat mix

- Straw pellets

Other: __________________________

2.1, 2.4, 2.7, 2.10 Is there visible recreational bedding material in the pen?

- Plenty

- Evidence of bedding

- Almost none

2.2, 2.5, 2.8, 2.11 How many pigs are in the pen?

2.3, 2.6, 2.9, 2.12 How old in weeks are the pigs in the pen?
