# Supplementary material for: Prevalence of human pathogenic Yersinia enterocolitica in Swedish pig farms
Source: Acta Vet Scand. 2018 Jun 25;60:39. doi: 10.1186/s13028-018-0393-5 (PMC6020225; doi:10.1186/s13028-018-0393-5)
Supplement: Supplementary file 3 — Additional file 3. A map showing the pig herds sampled per county. The distribution of samples reflects the non-uniform distribution of Swedish pig farms. [file 13028_2018_393_MOESM3_ESM.docx]

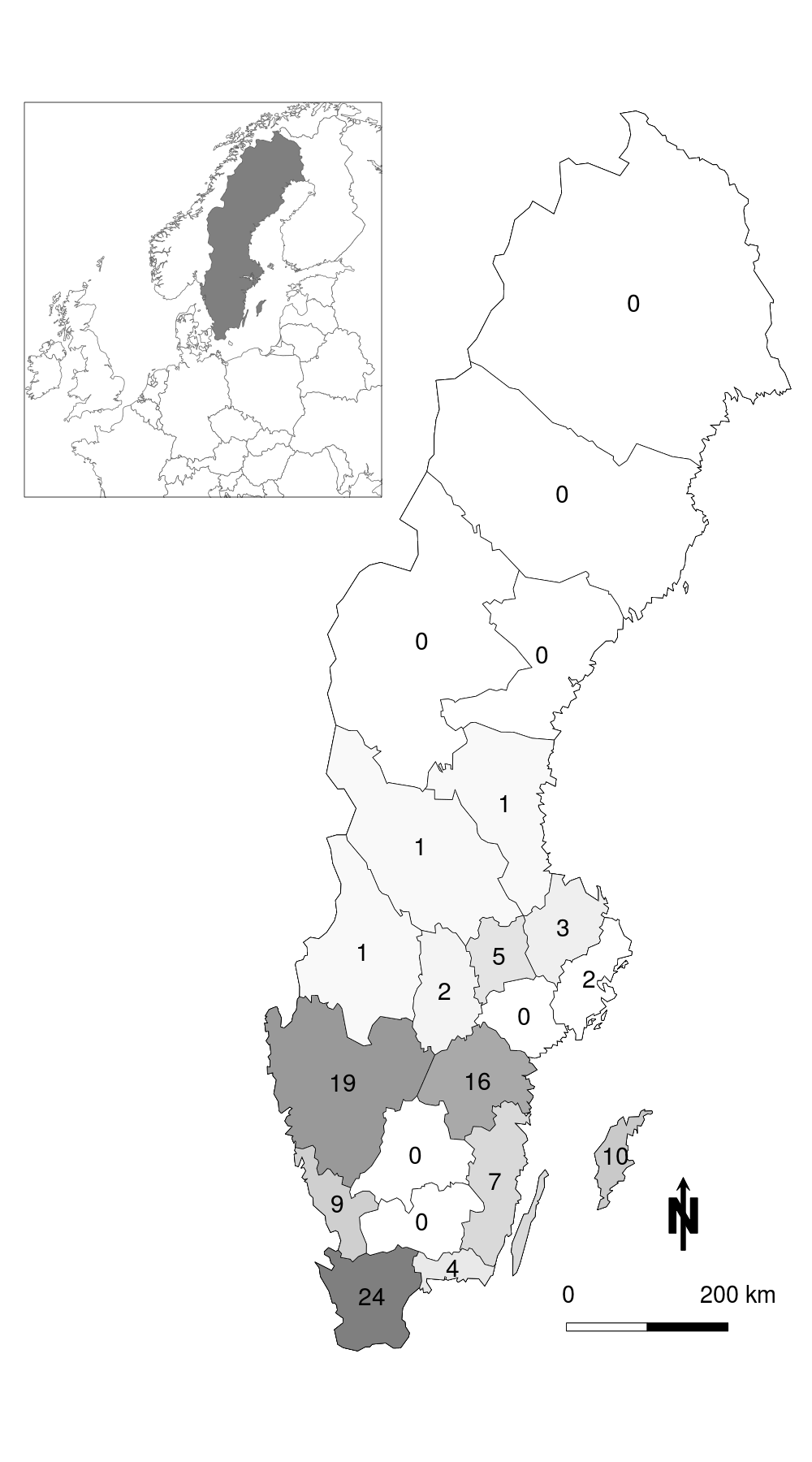


Additional file 3. A map showing the pig herds sampled per county. The distribution of samples reflects the non-uniform distribution of Swedish pig farms.
