# Supplementary material for: Prevalence of human pathogenic Yersinia enterocolitica in Swedish pig farms
Source: Acta Vet Scand. 2018 Jun 25;60:39. doi: 10.1186/s13028-018-0393-5 (PMC6020225; doi:10.1186/s13028-018-0393-5)
Supplement: Supplementary file 4 — Additional file 4: Appendix Table S1. The proportion of herds positive for Yersinia enterocolitica for each of the categorical variable levels recorded in the questionnaire. The P-values and associated odds ratios for the association of each variable with pen level Y. enterocolitica status, tested by logistic regression controlling for repeated pen measurements within herd by a random effect. [file 13028_2018_393_MOESM4_ESM.docx]

Additional file 4. Appendix Table S1.: The proportion of herds positive for *Y. enterocolitica* for each of the categorical variable levels recorded in the questionnaire. The *P*-values and associated odds ratios for the association of each variable with pen level *Y. enterocolitica* status, tested by logistic regression controlling for repeated pen measurements within herd by a random effect.

| Variable from questionnaire | Level | *Y. enterocolitica* Status | OR (95% CI) | *P*-value |
| --- | --- | --- | --- | --- |
| **Herd type** | Breeding | 2/5 | - | Ref |
|  | Gilt producer | 0/1 | - | 1.00 |
|  | Farrow to finish | 8/27 | - | 0.33 |
|  | Centre node of a sow pool | 1/1 | - | 0.879 |
|  | Satellite node of a sow pool | 1/18 | - | 0.131 |
|  | Finisher only herd | 20/52 | - | 0.401 |
|  |  |  |  |  |
| **Pig flow** | Always empty pens | 29/90 | - | Ref |
|  | Most often empty pens + Never | 3/12 | - | 0.79 |
|  |  |  |  |  |
| **Feed type** | Wet | 31/97 | - | Ref |
|  | Dry | 1/5 | - | 0.675 |
| **Feed type** | Added Whey + | 11/32 | - | 0.94 |
|  | No added whey | 21/69 | - | Ref |
| **Feed type** | Complete feed | 5/14 | - | 0.866 |
|  | No Complete feed | 27/86 | - | Ref |
|  |  |  |  |  |
| **Outdoor access** | Yes | 2/104 | - | - |
|  | No | 102/104 | - | Ref |
|  |  |  |  |  |
| **Evidence of birds** | Greater than Seldom | 3/9 | - | Ref |
|  | Never/Seldom | 29/93 | - | 0.9662 |
|  |  |  |  |  |
| **Evidence of rodents** | Greater than Seldom | 16/47 | - | Ref |
|  | Never/Seldom | 16/55 | - | 0.55 |
|  |  |  |  |  |
| **Type of flooring in finisher pens** | Solid floor with gutter | 0 | - | - |
|  | Slatted + solid floor | 31/97 | - | - |
|  | Deep litter or outdoor pens | 0/2 | - | - |
|  |  |  |  |  |
| **Type of recreational bedding material** | Straw | 22/70 |  | Ref |
|  | Straw and Wood shavings | 9/28 | - | 0.987 |
|  | Straw, Wood shavings and peat | 0/2 | - | 1 |
|  | Straw pellets | 1/1 | - | 0.442 |
|  |  |  |  |  |
| **Pen cleaning methods -** Multivariable logistic regression model | Scraping Yes; No | 13/37; 19/64 | - | 0.22 |
|  | High pressure (cold) Yes; No | 27/62; 5/39 | 84.77 (4.05-1772) | 0.00421 |
|  | High pressure (hot) Yes; No | 8/36; 24/65 | - | 0.11 |
|  | Detergent Yes; No | 2/5; 30/96 | - | 0.38 |
|  | Disinfectant | 14/43; 18/58 | - | 0.49 |
|  |  |  |  |  |
| **Pen drying** | Drying period Yes; No | 8/35; 24/66 | - | 0.12 |
