# Supplementary material for: Prevalence of human pathogenic Yersinia enterocolitica in Swedish pig farms
Source: Acta Vet Scand. 2018 Jun 25;60:39. doi: 10.1186/s13028-018-0393-5 (PMC6020225; doi:10.1186/s13028-018-0393-5)
Supplement: Supplementary file 5 — Additional file 5: Appendix Table S2. A summary of the continuous variables recorded in the questionnaire and the P-values and the associated odds ratios for the association of each variable with pen level Y. enterocolitica status, tested by logistic regression controlling for repeated pen measurements within herd by a random effect. [file 13028_2018_393_MOESM5_ESM.docx]

Additional file 5. Appendix Table S2: A summary of the continuous variables recorded in the questionnaire and the *P*-values and the associated odds ratios for the association of each variable with pen level *Y. enterocolitica* status, tested by logistic regression controlling for repeated pen measurements within herd by a random effect.

| Continuous variable | Description | Summary | OR | *p*-Value |
| --- | --- | --- | --- | --- |
| **Herd size*** | Number of pigs slaughtered per year | Min = 623, 1^st^Q = 2672, Med = 4235, Mean = 5625, 3^rd^Q = 6826, Max = 39944 | - | 0.61 |
| **Number of pigs in sampled pen** | count | Min = 3, 1^st^Q = 8, Med = 9, Mean = 9.47, 3^rd^Q = 10, Max = 50 | - | 0.58 |
| **Age of pigs in sampled pen** | Age in weeks | Min = 9, 1^st^ Q = 20, Med = 22, Mean = 20.92, 3^rd^ Q = 23, Max = 26 | - | 0.59 |

* Herd size was also tested on the log scale and with a squared interaction term. No significant associations were identified.
